# In human–machine trust, humans rely on a simple averaging strategy

**DOI:** 10.1186/s41235-024-00583-5

**Published:** 2024-09-02

**Authors:** Jonathon Love, Quentin F. Gronau, Gemma Palmer, Ami Eidels, Scott D. Brown

**Affiliations:** Psychological Sciences, University of Newcastle, University Drive, Callaghan, NSW 2308 Australia

**Keywords:** Human–machine team, Trust, Automation, Judge–advisor system

## Abstract

With the growing role of artificial intelligence (AI) in our lives, attention is increasingly turning to the way that humans and AI work together. A key aspect of human–AI collaboration is how people integrate judgements or recommendations from machine agents, when they differ from their own judgements. We investigated trust in human–machine teaming using a perceptual judgement task based on the judge–advisor system. Participants ($n=89$) estimated a perceptual quantity, then received a recommendation from a machine agent. The participants then made a second response which combined their first estimate and the machine’s recommendation. The degree to which participants shifted their second response in the direction of the recommendations provided a measure of their trust in the machine agent. We analysed the role of *advice distance* in people’s willingness to change their judgements. When a recommendation falls a long way from their initial judgement, do people come to doubt their own judgement, trusting the recommendation more, or do they doubt the machine agent, trusting the recommendation less? We found that although some participants exhibited these behaviours, the most common response was neither of these tendencies, and a simple model based on averaging accounted best for participants’ trust behaviour. We discuss implications for theories of trust, and human–machine teaming.

## Introduction

The last decade has seen remarkable advances in the development and use of Artificial Intelligence (AI) across a range of fields leading experts to conclude that AI will replace humans in a growing number of areas (Grace et al., 2018).

Although AI can outperform humans in many tasks, there remain areas where AI either does not perform as well as humans, or where AI performance is different from humans—that is, humans outperform AI in some portion of the task, and AI outperforms humans in some parts of the task (Hemmer et al., 2024). For example, Tejeda et al. (2022) demonstrated an image classification task where humans outperform AI on some images, and AI outperforms humans on others. In these instances, humans and AI working together can achieve better performance than either could on their own, be it in accuracy or speed or both. For this reason, there is growing interest in “human–bot teaming” for domains where high-quality decisions are important.

A crucial component to this human–machine teamwork is the exercise of trust. If the human fails to trust the bot sufficiently, they may ignore or not sufficiently incorporate an accurate bot’s recommendation into their final decision. Conversely, if the human comes to over-trust the bot, relying on it greater than is warranted, then once again, the human–bot team will fail to achieve its potential. Trust must therefore be appropriately *calibrated* (Lee and Moray, 1994; Muir, 1987).

Many studies have investigated this *calibration*, by having participants complete a task with the help of a machine agent. These typically involve the participant making a judgement, receiving a recommendation or advice from the agent, before having the opportunity to update their judgement (i.e. Yu et al., 2017). The degree, or the frequency with which the participant shifts their judgement in favour of the agent is taken as an indication of trust.

The present study had participants complete a task with the assistance of a machine agent. Unlike previous studies that typically have participants complete a small number of very different judgements, we make use of a simple perceptual task with a large number of trials. Such an approach allows us to avoid aggregating across participants, allowing us to draw inferences about trust behaviour at the level of the individual, and allowing us to investigate individual differences in trust behaviour.

We use this approach to investigate two aspects of human trust behaviour; whether humans rely on stable or varying trust strategies in machine assisted tasks, and the effect of *advice distance*—the distance between a person’s initial judgement, and the recommendation or advice they received—on people’s tendency to trust advice and incorporate it into their final judgement.

### Background

#### Trust

A dominant definition of trust in the literature was offered by Lee and See (2004), suggesting it can be viewed as “the attitude that an agent will help achieve an individual’s goals in a situation characterised by uncertainty and vulnerability”. Trust has historically been primarily measured in one of two ways, self-report (i.e. having participants respond to a Likert item “I trust the agent”), and through behavioural measures, (i.e. how much the participant uses an automation aid, or the proportion of time that a person changes their judgement in the face of a contradicting judgement from an aid).

Although some studies found a strong relationship between self-report and behavioural measures (Yu et al., 2018), other studies have demonstrated considerable dissociations between self-reported trust and trust behaviour. For example, Wiegmann et al. (2001) found that estimates of the reliability of a decision aid (what they characterise as a measure of *subjective trust*) dissociated from participant’s usage behaviour, leading them to question whether self-reported trust tapped the same construct as trust behaviour. Similarly, Schmitt et al. (2021) had participants complete multiple choice comprehension questions with the aid of a conversational bot, and found substantial differences between self-reported measures of trust and trust behaviour. This led them to coin the term “trust reliance paradox”. These findings echo similar findings of differences between self-report and behavioural measures found in investigations of inhibitory control (Snyder et al., 2021; Wennerhold & Friese, 2020).

The studies above suggest that self-reported trust, at times, may not be a very good predictor of human behaviour. This is significant, because in many domains it is ultimately behaviour, or the decisions made, that are more important than what a person may self-report, or how they feel about a decision aid. For example, consider a radiographer aided in the diagnosis of a cancerous tumour. The diagnosis they make, which ultimately leads to the prescribed treatment, is arguably far more important than what they might report about the aid. For this reason, the present study focuses on behavioural measures.

#### Human–machine trust

Many studies have investigated the way that humans come to trust or distrust machines or AI. These studies typically involve performing a task with assistance from a recommendation system, and coming to learn both their own performance and that of the agent. For example, Yu et al. (2017) had participants classify items coming from a production line as either faulty, or not faulty. Assisting the participants was an aid that would provide its judgement as to whether the item was faulty or not. The participants could accept the aid’s advice (or not) in making a final decision. After the decision, the true state of the item was revealed, allowing the participant to get a sense of their own performance and the aid’s.

This approach has been used to investigate a range of phenomena, including scenarios involving humans working with machinery to detect manufacturing faults (Yu et al., 2018), a satellite imagery search task with a helpful agent (Blaha et al., 2020), a simulated item classification task aided by a swarm of AI drones (Hussein et al., 2020), predicting the outcome of speed-dating couples (Yin et al., 2019), and judging whether product reviews are genuine or fraudulent (Schemmer et al., 2022). Perhaps unsurprisingly, studies have demonstrated that people are more willing to trust a machine agent that is more accurate, than a less accurate machine agent.

However, perhaps the most interesting findings are in the way participants weigh their responses against those of the machine agent. For example Dzindolet et al. (2003) had participants make judgements about whether a photograph contained a camouflaged soldier, before receiving a judgement or recommendation from a decision aid. Participants could then use either their initial judgement, or that of the aid. Although participants were less accurate than the decision aid, participants did not come to trust the decision aid, preferring to use their own judgement. Similarly, Dietvorst et al. (2015) had participants complete a forecasting task where the participant would forecast, and then receive a forecast from an algorithm. The participant could then choose between using the algorithmic forecast (an act of trust), or use their own forecast instead (distrust, or greater trust in themselves). Dietvorst et al. found that participants, after seeing the algorithm make a mistake, lost greater trust than participants who saw the same mistake committed by a human. These findings have been interpreted as people exhibiting ’algorithm aversion’.

Such studies have shed considerable light on human biases in human–machine teaming; however, a common feature to these studies is that the tasks involve participants responding to a dichotomous item; they can either trust the agent, or they can elect not to. This forces the participant to go one way or the other, and fails to discriminate whether the participant is experiencing a very high level of trust, or perhaps a more middling amount of trust. To some extent, the amount of trust can be assessed by aggregating across multiple trials, looking at the proportion of time that a person elected to trust the agent. However, this still fails to capture some important aspects of trust behaviour; for example, how trust varies from trial to trial. In this way, dichotomous measures likely overlook the finer points of trust. An alternative approach is to have participants respond on a *continuous* scale indicating trust. One such approach is that of the Judge–Advisor System.

#### Judge–advisor system

The judge–advisor system has been used to explore the way that people integrate advice from human “advisors”. It commonly involves asking participants to make some sort of judgement of a continuous quantity, such as estimating the year when the Suez Canal first opened (Yaniv, 2004), estimating the price of backpack models based on information about their features (Sniezek et al., 2004), estimating the mean annual salary of graduates from different business schools (Soll & Larrick, 2009), or estimating a person’s weight from a photograph (Gino and Moore, 2007). While initial applications focussed on advice from other human agents, more recently the judge–advisor system has been applied to situations where humans receive advice from AI aids (Logg et al., 2019).

Following their initial judgement, participants are exposed to the judgements of others, humans or AI, before updating (or not updating) the initial judgement. The extent to which participants revise their estimate towards the advisor’s estimate provides a measure of *advice taking*, something that can be understood as a behavioural measure of trust. Significantly, the degree to which a person changes their judgement (i.e. how far they move their second response in the direction of the advice) represents a continuous measure of trust. Such an approach provides a much richer account than the dichotomous *trust*, *do not trust* responses previously described.

#### Weight of advice

Historically, judge–advisor studies have computed the *weight of advice* as an index of trust behaviour (Harvey & Fischer, 1997; Yaniv, 2004). The weight of advice is calculated for each trial as follows:

1 $$\begin{aligned} {B - A} \over {R - A} \end{aligned}$$

where *A* denotes the participant’s initial response, *R* denotes the recommendation, and *B* denotes the participant’s second response (Harvey & Fischer, 1997; Yaniv, 2004). If the participant’s second response is the same as their first, $B - A$ = 0, and hence the weight of advice is zero. If the participant’s second response, *B*, is the same as the recommendation, *R*, the numerator and the denominator are equal, and weight of advice is one. Similarly, if the participant’s second response is half way between their first response and the advice, the weight of advice is 0.5. In this way, the weight of advice captures the degree to which a participant weighs their judgement against the advice given.

Judge–advisor studies typically report a mean *weight of advice* of 0.3 (Harvey & Fischer, 1997; Lim & O’Connor, 1995; Yaniv, 2004; Yaniv & Kleinberger, 2000), showing participants tend to favour their own judgement over that from third parties. However, Soll and Larrick (2009) observed that simply reporting the mean weight of advice may give the impression that participants are simply responding with a weighted average between their first response and the advice, when in fact they may be using a mixture of strategies. For example, a participant may elect to *stay* (i.e. not move their judgement; weight of advice of zero) 70% of the time, and *adopt* (i.e. completely adopt the recommendation; weight of advice of one) 30% of the time; this would lead to a mean weight of advice of 0.3, however the participant is never actually averaging. To investigate this possibility Soll and Larrick visualised weight of advice using a histogram, and found participants behaviour exhibited a tri-modal distribution with peaks at the values of 0, 0.5, and 1. This suggests that participants are using a mixture of strategies; on some trials they are not shifting from their first judgement (the peak at zero), on other trials, they are entirely adopting the advice (the peak at one), and some other proportion of trials, perhaps using some sort of weighted average (the values falling between zero and one). In this way, it is important to evaluate not just the weight of advice, but to visualise the distribution of weight of advice as well.

Although visualising the weight of advice provides insights into trust behaviour, there are limitations to what it tells us. Although it allows us to investigate the variation in participant strategies, it does not provide insight into what is driving those different strategies. For example, it might be that participants find advice falling closer to their initial judgement as more trustworthy, leading them to *adopt* the recommendation in full. Or it might be that distant recommendations lead the participant to doubt the agent, leading them to stay, not moving from their initial judgement. A means to investigate these potential strategies is to examine the effect of ’Advice distance’ on people’s willingness to trust.

#### Advice distance

The judge–advisor paradigm allows to investigate the effect of *advice distance*—the distance between the participant’s initial judgement, and the advice they receive—on trusting behaviour. For example, a participant may consider recommendations falling a long way from their initial judgement as implausible and untrustworthy, leading them to move their second response proportionally less. Alternatively, a participant having received a recommendation falling far from their initial judgement, may come to doubt their initial judgement, leading them to move their second judgement proportionally closer to the recommendation. In this way, exploring the effect of *advice distance* on “advice taking” could be quite revealing.

A number of studies have investigated this using the judge–advisor approach. Yaniv (2004) found that participants were more persuaded by advice that was closer to their initial estimate (aka *agent doubt*), than advice that was further from their initial estimate. In contrast, Mesbah et al. (2021) found the opposite finding, that more distant advice is given more weight (*self-doubt*), whereas Logg et al. (2019) found no effect of advice distance on people’s willingness to shift their judgement.

These different findings likely reflect different properties of each of these tasks slightly favouring different advice taking strategies. However, one aspect that may serve to obscure the picture each of these studies provides us is that all these studies aggregated data across all participants. Such aggregation could obscure the variation across participants in their advice taking. For example, different participants may adopt different strategies (e.g. averaging, *self-doubt*, *agent doubt*), that may not be identifiable after aggregation. Typical judge–advisor studies require aggregation because they contain a small number of items, and the items are, arguably, qualitatively different from one another. For example, Yaniv (2004) had participants make 15 judgements as to the year that historic events took place in the last 300 years. The small number of items, and the (likely) high variation in different participants’ knowledge of each of these events makes it hard to draw inferences at the level of the individual. Instead, it requires the experimenter to aggregate across participants, obscuring what might be interesting individual differences.

It may be that all three studies had participants exhibiting *self-doubt*, *agent doubt* and averaging, and the different findings may reflect different proportions of participants using each of these strategies. Once aggregated, the dominant strategy may have come to overpower the lesser used strategies, leading to the conclusion that these strategies were not used.

### Present study

The present study aims to investigate more closely the strategies that participants use, examining both the distribution of weight of advice, and the effect of advice distance on weight of advice. Crucially, we have participants complete a large number of homogenous perceptual judgements, providing enough data that aggregating across participants is not necessary. This allows us to investigate both strategy and the effect of advice distance, at the level of the individual.

#### Experimental task

**Fig. 1:**
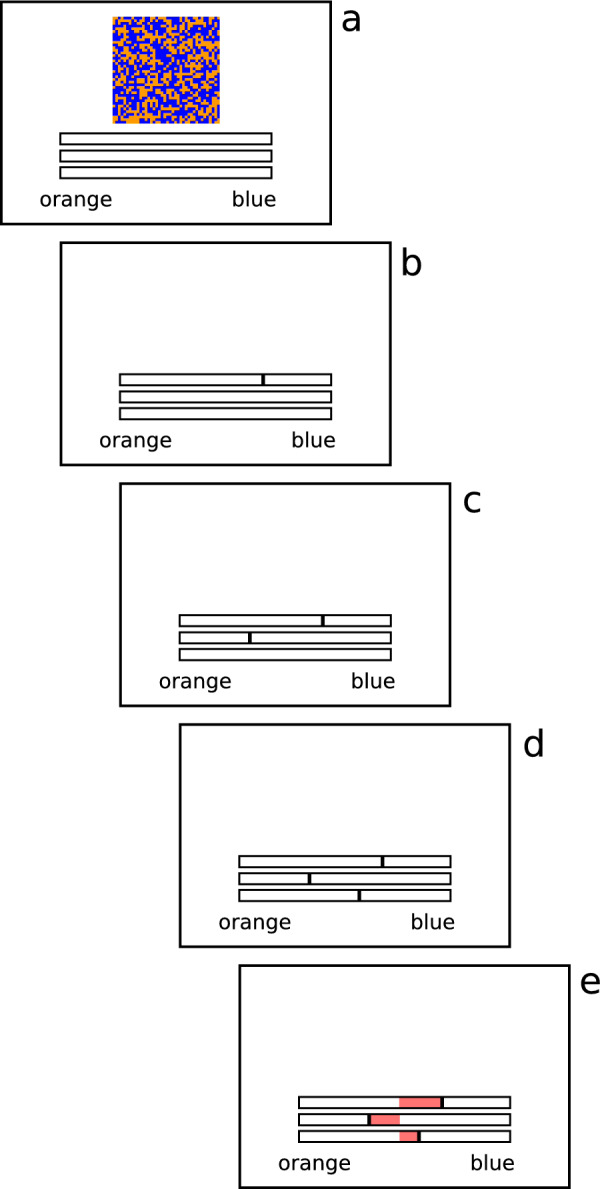
A trial sequence. **a** The moving dot stimulus is presented. **b** The participant indicates the proportion of blue and orange by responding on the first scale. **c** The participant receives a recommendation on the second scale. **d** The participant responds a second time, taking into account (or ignoring) the recommendation. **e** The true proportion is revealed, and the distance between the first response, the recommendation, and the second response is shaded. A large shaded area indicates an inaccurate judgement, a small shaded area indicates an accurate one

In our experiment, we used the judge–advisor system with people performing simple perceptual judgements. Specifically, participants were presented with rectangular arrays filled with blue and orange pixels, and had to provide estimates of the relative proportion of each colour, extending recent work from Love et al. (2023). The trial sequence is shown in Fig. 1. Participants view a display made up of orange and blue pixels (a) and then respond using a horizontal slider scale with an estimate about the proportion of colours (b). The participant then receives a recommendation from a bot, indicated on the second scale (c). The participant then responds a second time, updating their initial estimate after taking (or not taking) into account the recommendation (d). After this, feedback shows the accuracy of the participant’s responses and the bot recommendation (e). Across repeated trials, this feedback helps the participant develop a sense of their own expertise and the accuracy of the recommendations.

#### Strategy visualisation

In order to visualise each participant’s response strategies, we plot the *shift*, that is, how far the participant moves their response having seen the recommendation, against *advice distance*; the distance between the participant’s first response, and the recommendation. These terms are calculated as follows:

$$\begin{aligned}{} & {} Shift = { B - A } \\{} & {} Advice\ distance = { R - A } \end{aligned}$$

**Fig. 2:**
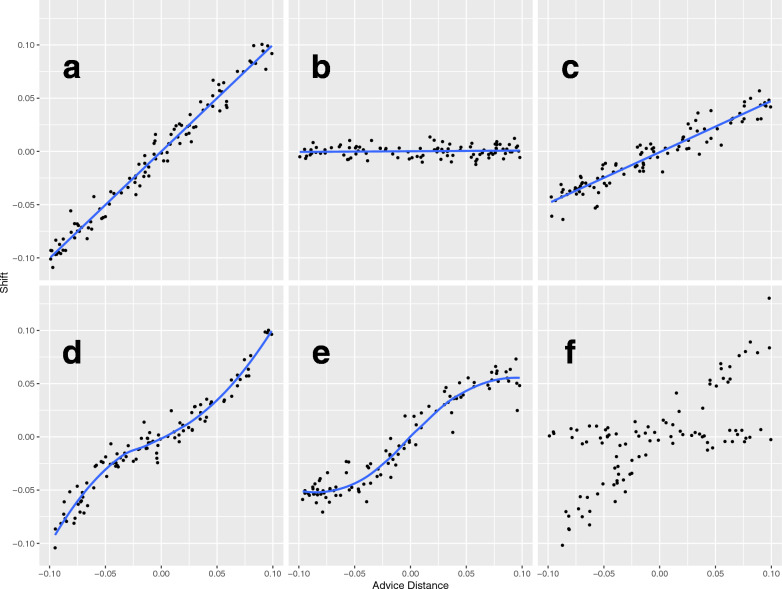
Example response strategies (simulated). **a** *Adopt*, the participant adopts the recommendation (gradient = 1). **b** *Stay*, the participant ignores the recommendation (gradient = 0). **c** *Compromise*, the participant compromises between their judgement and the recommendation (in this case, gradient = 0.5). **d** *Compromise with self-doubt*, the participant compromises, but considers distant recommendations to be more reliable. **e** *Compromise with agent doubt*, the participant compromises, but considers distant recommendations to be less reliable. **f** A combination of strategies, in this case the participant adopts on some trials, and stays on others

Plotting shift against advice distance separately for individual judgement trials may reveal different behaviours, as illustrated in Fig. 2. In each panel, the *x*-axis represents the *advice distance*, or the distance between a participant’s initial response, and the recommendation. If the recommendation falls to the right of the participant’s first response, then this value would be to the right of the origin [0, 0]. If the recommendation falls to the left of the participant’s first response, then this value would be to the left of the origin. The *y*-axis shows the shift that the participant makes between their first and second responses.

Figure 2 illustrates hypothetical results predicted by various response strategies. If the participant adopts the recommendation in full and makes their second response identical to the recommendation, then this would look like panel (a), a diagonal line with a gradient of 1. In contrast, if the participant completely ignores all recommendations, and does not shift between their first and second responses, this would look like panel (b), a horizontal line with gradient zero. Panel (c) shows a hypothetical participant who averages their response with the recommendation. In this example, the participant is averaging 50/50, yielding a gradient of 0.5, but of course a continuum of values are possible between 0 and 1. Gradients closer to 1 indicate greater trust, and gradients closer to zero indicating less trust.

Responses illustrated by panels (d) and (e) are similar to response type (c), however they describe the situation where a participant might trust recommendations either more or less, the further they land from their initial response. Panel (d) depicts the scenario whereas the recommendation falls further from their initial response, the participant doubts their initial judgement. Panel (e) depicts a different response, where a distant recommendation leads the participant to doubt the agent, trusting themselves more. Finally, panel (f) depicts a hypothetical participant who uses a mixture of strategies (as described in Soll & Larrick, 2009); on some trials they rely exclusively on their own initial belief without taking advice, and on other trials they rely exclusively on the advice.

## Methods

44 participants were recruited from The University of Newcastle first year student cohort, with a further 45 participants recruited via Prolific, giving 89 participants over all. The task took around 25 min. Participants recruited from university were compensated with course credit, and those recruited via Prolific were compensated with £3. Post hoc analysis examining differences across the two cohorts, which we report in more detail later and in the Appendix, showed on campus participants were slightly more accurate than those recruited via prolific but manifested the same qualitative patterns.

The trial sequence has been previously described and is depicted in Fig. 1. Colour proportions were drawn from a uniform distribution, resulting in proportions of blue and orange varying between 40 and 60%. The response scales were slightly wider, spanning 35 to 65% to allow the recommendation to fall outside symmetrically about the true proportion.

Recommendations were generated from a uniform distribution centred on the true value of the stimulus proportion in each trial. During the first phase of the experiment (*pre*) the recommendations were accurate, falling within 1.5% (within one 20th of the 35–65% scale). In the second phase (*adjustment*) the accuracy of the recommendations was degraded, falling within 10% of the true proportion (within one third of the 35–65% scale). In the third (*post*) phase, recommendations returned to the same accuracy as for the first phase.

Participants were not explicitly told where the recommendations were coming from (i.e. from another person, or from the computer), however the fact that the task was delivered online, and that the recommendations were delivered with perfectly consistent timing, participants would have inferred (correctly) that the recommendations were being generated by the computerised task they were completing.

Participants completed 110 trials in total. The first 5 trials simply acquainted participants with the judgement task, and provided no recommendations, nor solicited second judgements. Following these trials, the recommendations were introduced, and for the remaining 105 trials participants were solicited for second judgements.

In 20% of these trials, dispersed throughout the experiment, no recommendation was provided, and the participant was simply prompted to respond a second time. These trials allowed us to assess whether any observed improvement in participants’ second responses was due to receiving the recommendation, or if they improved simply by being asked to provide a second response. Love et al. (2023) also found that the presence of these no recommendation trials discouraged participants from issuing rushed, low quality, initial responses.

In pursuit of a separate line of enquiry, the onset of the adjustment phase was manipulated between subjects: for different subjects, the adjustment phase fell between trials 21–40, 41–60, or 61–80. This manipulation had no reliable effects on the data, and so we do not report it further. Readers interested in more detail can find full accounts of this manipulation, and inferential tests of its effects, in https://osf.io/z7erx/?view_only=cf89ec3903b44c67a147fecf65fb2f58.

## Results

Several software packages were used to analyse the data. Unless otherwise specified, data was prepared and analysed in jamovi (The jamovi project, 2023), with Bayes factors provided by BayesFactor (Morey & Rouder, 2022) and JASP (Love et al., 2019).

As a first check that participants understood the task and followed instructions, we tested that participants’ first and second responses were sensitive to the ground truth of the stimulus, and also that their second responses were closer to ground truth (i.e. more accurate) than their first responses, on average. Both of these checks were supported by the data. The correlation between ground truth stimulus colour balance and participants’ responses, over all trials and participants, was $r=.791$ for first responses and $r=.897$ for second responses (both significantly different from zero, according to both frequentist and Bayesian tests, $p<.001$, $BF>10^6$).

Next, for each trial we calculated *deviance* scores for the participant’s first and second responses, as the absolute difference between each response and the ground truth. A value of zero indicates a perfect judgement, and a higher value indicates a less accurate response. We then took the average of each of these values for each phase for each participant, and then took an average of these. These results, along with deviance values for the recommendations appear in Table 1. Further detail, including the number of trials going into each calculation, and the performance of the on campus vs online recruits is reported in the Appendix.

**Table 1:** Mean of participants’ means of *Deviance* (an indicator of accuracy) for the first response (A), the recommendation (R), the second response (B), and the *Weight of advice*, for each of the three phases of the experiment

Phase	Deviance			Weight of Advice
	1st Response (A)	Recommendation (R)	2nd Response (B)	
Pre	3.06	0.75	1.58	0.57
Adjusted	2.91	5.00	2.75	0.32
Post	2.75	0.75	1.79	0.46

As can be seen, the recommendations in the *pre* and *post* phase were more accurate than participants, but less accurate in the adjusted phase.

### Weight of advice

For each trial, weight of advice was calculated using equation 1. We employed a convention from the judge–advisor literature to truncate, or *winsorize* weights of advice; to treat values less than zero as zero, and values greater than one as one (Soll & Larrick, 2009; Himmelstein, 2022).

**Fig. 3:**
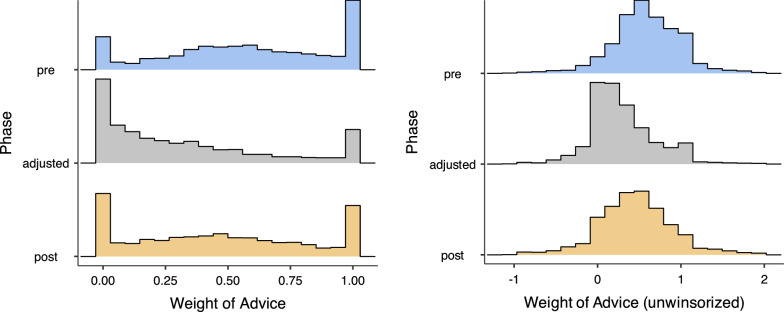
Histogram of all weights of advice, collapsed across participants. The left panel represents the standard approach where values are *winsorized*; values less than zero are treated as zero, and values greater than one are treated as one. The right panel depicts the raw, unwinsorized values

As can be seen in Table 1 and Fig. 3, participants were sensitive to the accuracy of the recommendation. Participants responded to the reduced accuracy in the adjustment phase by reducing the weight they put on the recommendation. When the accuracy of the recommendation was restored in the *post* phase, participants placed greater weight on the recommendation. However participants did not weigh recommendations in the *post* phase as high as they did in the *pre* phase, even though the recommendations were the same accuracy. It appears the low accuracy *adjusted* phase had some enduring effect. A repeated measures ANOVA on weight of advice confirmed that all three phases were significantly different from each other ($p <.001$, $BF > 10^6$).

An alternative explanation is that due to practice effects, participant performance on the first judgement improved over the course of the experiment, such that they no longer needed to rely so much on the recommendation. Consistent with this explanation, participants’ initial judgements in the *post* phase were more accurate than those in the *pre* phase. Although this might account for some of the apparent reduction in trust, participants also performed decidedly worse on their second judgements in the *post* phase than in the *pre* phase. This suggests that the reduction in trust was disproportionate to their increase in ability, and reflects enduring reduced trust. Relying less heavily on the recommendation harmed their performance, rather than improved it.

Figure 3 shows histograms of the *weight of advice* of every trial in each phase, collapsed across all participants. Figure 3, left panel, displays histograms for the winsorized values. As can be seen, there is some evidence of the tri-modality identified by Soll and Larrick (2009). Trials in which the recommendation was ignored (zero weight of advice) or completely adopted (unit weight of advice) are over-represented. However, if we consider the right panel of Fig. 3, we can see that these peaks at zero and one are in fact artefacts of the winsorizing process, and do not actually occur in the data.

These histograms collapse over participants, so it is impossible to know whether these effects are due to different participants using different strategies, or participants changing strategies across trials. In the next section, we investigate individual-participant strategies in more depth.

### Advice distance and trust

**Fig. 4:**
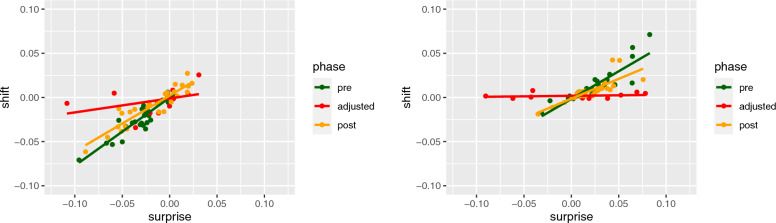
Examples of participant data—plotting *shift* against *advice distance*. Both participants exhibit high *weight of advice* in the pre phase (green line), with gradients close to one. In the adjusted phase, participants respond to the less accurate recommendations with a reduced weight of advice, closer to zero. In the final phase, participants’ weight of advice increases again

For each participant, we plotted *shift*, how far the participant shifted their second response from their first response ($B-A$), against *advice distance*, how far the recommendation fell from their first response ($R-A$). Figure 4 shows these plots for two example participants. Note that we discarded the first 10 observations from the experiment, and the first 5 observations from the second and third phases, to allow some time for adjustment. For these two participants an averaging account, represented by a straight line, accounts for the data quite well (cf. Fig. 4), as was the case with most participants; there was little to suggest that participants made use of a mix of strategies or that they shift more (doubting themselves) or shift less (doubting the recommendation), when the recommendation is very surprising.

We formally tested these intuitions across all participants via Bayesian model selection. Three models were fitted to each participant’s data from each of the three phases. The three models capture the different response strategies illustrated earlier with Fig. 2. One model assumed a linear relationship between shift and advice distance, which can capture the three response strategies shown in panels (a)–(c) of Fig. 2. Another model assumed a linear relationship between shift and the square of advice distance (multiplied by the correct sign), which captures the self-doubt strategy shown in Fig. 2d. The third model assumed a linear relationship between shift and the square root of the absolute value of advice distance (multiplied by the correct sign), capturing the agent doubt strategy of Fig. 2e.

**Fig. 5:**
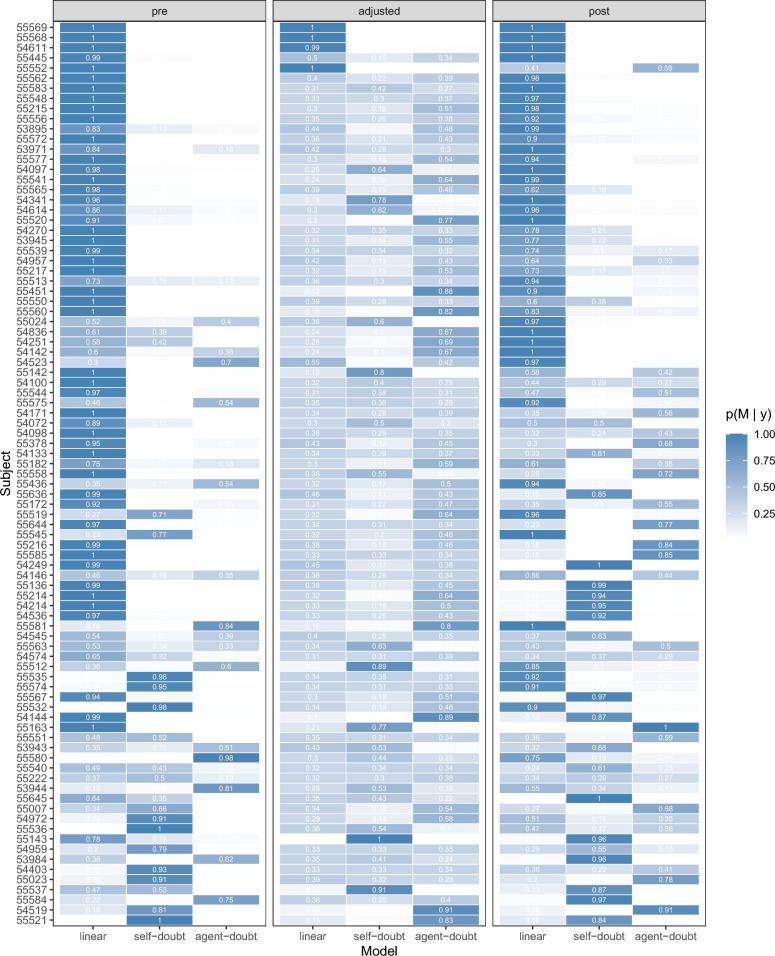
Posterior model probabilities for each of the three models, for each of the phases, for each participant. Posterior probabilities add to 1 for each person (row) within each phase of the experiment (pre, adjusted, and post). Rows are ordered by overall support for the linear model

We implemented all three models in rstan (Stan Development Team, 2023). We used the Jeffreys–Zellner–Siow linear regression prior set-up as implemented in the BayesFactor package (Morey & Rouder, 2022) with the exception that there was no prior on the intercept as it was not part of the models. For each participant, experiment phase, and model, we drew a total of 36,000 samples from the posterior distribution. Specifically, we ran four chains for 10,000 iterations and discarded the first 1,000 iterations of each chain as burn-in. Convergence of the chains was assessed using the $\hat{R}$ statistic which was below 1.01 for all samples (Brooks & Gelman, 1998).

We then compared the models in their ability to account for the data by estimating the marginal likelihood for each model using Warp-III bridge sampling (Gronau et al., 2020a, b). The marginal likelihood compares how well a model accounts for the observed data while also penalising for model complexity. Marginal likelihoods were used to calculate posterior model probabilities, which are shown in Fig. 5. These are based on assuming equal prior model probabilities for all three models. Each row in that figure represents one participant, and the three big columns represent the pre, adjustment, and post phases of the experiment. Within each big column there are three cells which are shaded between white (zero) and blue (one) showing the posterior model probability for the three models. Within each big column, the left-hand mini-column shows the probability that the participants are best described by the linear model, the centre mini-column shows the probability that the participants are best described by the agent doubt model, and the right-hand mini-column shows the probability that the participants are best described by the self-doubt model. Rows (participants) have been ordered from top to bottom by those most clearly identified as using the linear model in all phases to those least such.

The fewer trials in the *adjustment* phase, as well as the large changes in trust, made model identification less certain there, so we focus on the *pre* and *post* phases of the experiment. Table 2 summarises the counts of the preferred model for each participant in the *pre* and *post* phases. As can be seen, the linear model was the favoured account for many participants. Of the 89 participants, 36 favoured the linear model in both *pre* and *post* phases, and 77 favoured the linear model in at least one of those two phases.

A small number of participants exhibited behaviour consistent with a *self-doubt* model, with 16 favouring a self-doubt account in the *pre* phase, and 22 favouring a self-doubt account in the *post* phase. However, only 4 participants exhibited this behaviour consistently across both the *pre* and *post* phases.

The *agent doubt* model was the least favoured, with only 10 and 17 participants favouring the agent doubt model in the pre and post phases, respectively, and no participants favouring the agent doubt model in both phases.

**Table 2:** Paired contingency table showing counts for the preferred models for the *pre* and *post* phases

	*Preferred post*			*Total*
	Linear	Self-doubt	Agent-doubt	
*Preferred pre*				
Linear	36	15	12	63
Self-doubt	7	4	5	16
Agent-doubt	7	3	0	10
*Total*	50	22	17	89

### Trust without feedback

One of the key ways in which the paradigm departed from previous judge–advisor studies is its use of trial-by-trial feedback. Judge–advisor studies do not typically provide feedback, almost seeing if participants will take a “leap of faith” in trusting an advisor they often have no real reason to trust or doubt. There is one trial in our study that might be understood to offer participants a similar opportunity for a leap of faith; the very first trial of the adjusted block. This trial typically presents the user with a judgement considerably further from where they might expect the truth to lie. The participant is confronted with what must seem to be an implausible recommendation, but which comes from a source that, until now, has been very reliable.

To some extent this is true for all the trials in the adjusted phase, however the first trial is unique in that the participant must make a judgement before seeing any feedback that might confirm the recommendations inaccuracy. In this way, the very first trial of the adjusted phase represents something more closely aligned to traditional judge–advisor studies.

We compared weight of advice for participants on the trial immediately preceding the onset of the adjusted block, with the very first trial of the adjusted block. There was a slight decline in mean weight of advice (0.593 before, 0.499 after) however this was only just significant (Paired *t*-test, $t_{73} = 2.15, p=.034$) and the Bayes factor was equivocal ($BF_{10}=1.15$).

Even if we interpret this as a small decline in trust, participants seemed willing to take a reasonable “leap of faith”, given an aberrant recommendation. Consistent with this, we can see that the mean weight of advice for the first adjusted trial (0.499) is considerably higher than the mean weight of advice for all the trials in the adjusted phase (0.32, as seen in Table 1).

## Discussion

We had participants complete a perceptual task where they issued a pair of judgements about the proportion of colours in a display. The first judgement was performed unaided, and the second judgement was performed after receiving a recommendation on the true proportion from an agent. The degree to which participants shifted their response in favour of the recommendation is designated *weight of advice* and captures a behavioural measure of trust.

Consistent with previous literature, participants were sensitive to the accuracy of recommendations. Reducing the accuracy of the recommendation led to a marked decline in the weight participants were willing to put on the recommendation. When the accuracy of the recommendation was restored, in the *post* phase, participants regained trust but not to the level they originally held in the *pre* phase. A simple explanation here might simply be that trust, once lost, is difficult to gain. This finding is consistent with Yu et al. (2017), Yaniv and Kleinberger (2000), and is sometimes referred to as *trust asymmetry* (Poortinga & Pidgeon, 2004). Significantly, this effect has been demonstrated to be more pronounced in human–machine trust, with Dietvorst et al. (2015) finding that participants lose more trust, in response to low quality recommendations, when they believe the recommendations are coming from a machine agent rather than a human.

### Trust is quick to lose, slow to gain

Yu et al. (2017) examined the way that participants responded to automation failures and successes, and found trust declined more in response to automation failures, than it increased in response to successes. Similarly, Yaniv and Kleinberger (2000) employed the judge–advisor paradigm and assigned participants to one of two conditions involving either accurate, or inaccurate recommendations in the first block of the experiment. Subsequently, the quality of the recommendations, in both conditions, became similar (average). Yaniv and Kleinberger observed that participants’ weight of advice declined more from the accurate to average condition, than it increased from the inaccurate to average condition. Yaniv and Kleinberger took this as evidence that trust (or reputation, as they called it), is more easily lost than gained.

Our paradigm hints at a possible explanation for why this occurs. Highly trusting participants develop a mental model that recommendations fall close to the truth, and therefore have reason to doubt their mental model even if they see just one single recommendation fall much further from the truth. In contrast, un-trusting participants develop a mental model that recommendations are highly variable. For these participants, accurate recommendations (close to the truth) do not falsify their mental model in the same way, as even highly variable recommendations will be accurate some of the time. In this way, trust might be more easily lost than gained. This account holds considerable appeal, as it accounts for the asymmetry of gaining and losing trust through a simple cognitive process, rather than relying on a more anthropomorphic account.

### A continuous scale for weight of advice

Considering the distribution of weight of advice, our study replicated the finding of Soll and Larrick (2009), with histograms of weight of advice exhibiting clear peaks at zero and one, and something of a peak close to 0.5. However, if we consider the histogram of unwinsorized values, we see that the peaks at zero and one were almost entirely driven by the winsorizing, rather than from the data itself. This raises the question of whether the peaks in Soll and Larrick (2009) were also artefacts of winsorizing.

In our case, the peaks were driven by a large proportion of responses that fell outside the 0 to 1 range. Across all participants, 26% of responses were either less than zero or greater than one; whereas, Soll and Larrick (2009) only reported 4.6% of responses falling outside the zero to one range. The two peaks reported in Soll and Larrick (2009) were of magnitudes of around 10%, together, representing 20% of participant’s responses. As such, their peaks would have been largely driven by the data.

It may seem puzzling that such a large number of responses in our study fell outside this range, however this is likely a product of our continuous response scale. For example, a substantial number of recommendations will fall close to the participants initial response. The participant may then intend to issue a response either identical to their first, identical to the recommendation, or very close to either of these. However due to the imprecision of using a computer mouse (or what we might call *motor noise*), their second response may fall outside the zero–one interval. Consistent with this account, participants issued very few responses with weights of advice greater than one (6.9%) in the adjusted phase when the recommendations fell far from the truth, compared to the other phases (14.3%).

Our continuous scale is a marked departure from prior judge–advisor studies, where participants enter their judgements through entering numerical values. Such entry appears to side step the issue of motor noise altogether. This may seem superior to the continuous response scale, however it should be noted that there still may be signal in this noise, and that our models fitting *shift* against *advice distance* meaningfully incorporate this noise into the model’s estimates. In contrast, models that winsorize these extreme values discard information and likely introduce bias. Indeed, given the linear model’s ability to make use of weights of advice greater than one and less than zero, it may be a preferable to use the estimated gradient of such a model as a measure of advice taking, rather than the mean winsorized weight of advice.

### Advice-taking strategies

With regards to strategy, within a phase, many participants adopted a stable strategy responding fairly consistently with a weighted averaging strategy. This finding is at odds with the work of Soll and Larrick (2009), who found that participants typically use a mixture of strategies; *averaging* and *choosing* (*adopting* or *staying* were characterised as *choosing*). Soll and Larrick found that people were insufficiently sensitive to the conditions that favour averaging, leading them to use a *choosing* strategy when averaging would have been optimal. In contrast, we find participants making extensive use of averaging.

One obvious explanation is that our items were essentially the same judgement, with only the proportion of colour and the quality of the recommendation varying from trial to trial, whereas in Soll and Larrick, and indeed almost all of the judge–advisor literature, the items were heterogeneous, with each item being qualitatively different. The differences between the items may serve to provoke different response strategies from participants, in a way that our uniform task did not.

Another possibility is that heterogeneous strategies are driven by the absence of trial-by-trial feedback. Soll and Larrick (2009) did not provide participants with feedback after each item, and participants were likely left in a greater state of uncertainty as to the effectiveness of their strategies, perhaps leading them to select strategies with some randomness. It is possible that the heterogeneous strategies that Soll and Larrick observed are a product of a listlessness on the part of participants as they fail to find a reason to prefer one strategy over another. In contrast, the provision of feedback may allow participants to see that their strategy is effective, and so lead to stable response strategies.

### Trust and advice distance

Overall we found that a linear model accounted for the trust behaviour of the largest proportion of participants (see Table 2). A much smaller proportion of participants exhibited behaviour consistent with models that allowed participants to doubt either themselves, or the agent, in response to more distant recommendations. Additionally, some participants appeared to use different strategies in different phases of the experiment.

This casts some doubt on earlier studies that have aggregated judgments across participants and reached general conclusions about the effect of advice distance (i.e. Yaniv, 2004; Mesbah et al., 2021; Logg et al., 2019). It also provides some support for the idea that mixed findings from earlier studies may be a result of individual differences.

Many participants were not favoured by a consistent model across the *pre* and the *post* phase. This may reflect genuine shifts in strategy by participants, however it may also reflect challenges in the model fitting process. A clue to what may be happening here is that the model selection was more decisive in the *pre* phase, than in the *adjusted* or *post* phase. The *pre* phase had participants use recommendations that were stable and predictable in their accuracy. In contrast, the *adjusted* and the *post* phases both began with a significant shift in the accuracy of the recommendations. Participants were likely left in a state of considerable uncertainty as to the accuracy of the recommendations, and might have varied their strategy over the first few trials. This would then be reflected in poorer model fits in the *adjusted* and *post* phases. To tackle this issue, we excluded the first 5 trials from each of the phases when fitting the models, however, for a number of participants, it could have taken more than 5 trials to settle into a stable response strategy (such as averaging).

**Fig. 6:**
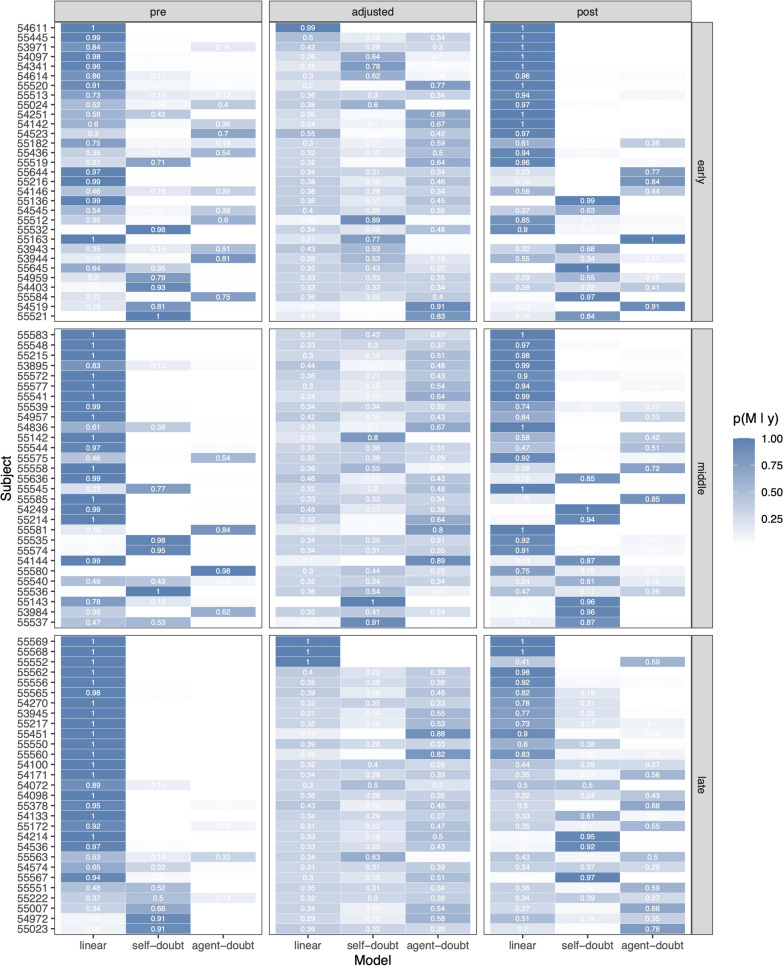
Posterior model probabilities for each of the three models, for each of the phases, for each participant, split by *Early*, *Middle*, and *Late* conditions. Other details are as for Fig. 5

Support for this conjecture is found in examining the model fits for participants when split by the *Early*, *Middle*, and *Late* conditions, as reported in Fig. 6 in the Appendix. The *Early*, *Middle*, and *Late* conditions were a parallel enquiry into the effect of varying the timing of the *adjusted* phase on participant trust. No difference was found, and the lack of findings have not been a focus of this paper. However, when splitting the model fits up by *Early*, *Middle*, and *Late* conditions, we observe clearer model selection in the *post* phase of the *Early* condition, and the *pre* phase of the *Late* condition. In both these cases, participants completed a larger number of trials—60 and 65, respectively—and exhibited much clearer model selection, and the favouring of a linear account.

The larger number of trials in these phases could have served to dilute the influence of the earlier trials, allowing more decisive model selection. In contrast, phases with fewer trials led to greater ambiguity in model selection. Regardless, the favouring of the linear model appears to be a robust finding, with many participants being favoured by the linear model in one or both phases.

### Future directions

A limitation of the present paradigm is that it can only capture “relative trust”. The *weight of advice* only captures something of the difference, or a ratio, between the participant’s trust in themselves, and the trust in the agent, but cannot inform about the participant’s trust in absolute terms.

One way in which this might be addressed in future research is by applying a cognitive model. It might be possible to model participants’ perception of their performance, and the perception of the agent’s performance with distributions. A wider distribution representing the judgements of a less accurate agent, and a narrower distribution representing more accurate ones. Such a cognitive model could shed light on the way people integrate the judgement of a trusted agent with their own judgements. Further, such a model would allow the assessment of discrepancies between the true distribution, and participants’ mental models, allowing the characterisation of bias in trust behaviour in a fine tuned way.

Perhaps more significantly, such a cognitive model would characterise trust in terms of perception of an agent’s performance. This allows precise quantitative predictions and measurement, which represents a more “bold hypothesis” in the sense defined by Karl (Popper, 1999). This contrasts with many current models of trust, most of which make the much more vague and less falsifiable prediction of a simple positive relationship between trust and trusting behaviour.

A final question of our findings is just how far these findings are likely to generalise to real world phenomena. As discussed, our task made use of highly homogenous perceptual judgements, whereas if we consider real world human–machine team scenarios such as radiography or cybersecurity, the judgements involved seem more varied and heterogeneous. How well our findings generalise to more heterogeneous tasks is a matter for future research.

In conclusion, in this paper we investigated trust of human users in AI advice using a perceptual paradigm allowing continuous measures of trust. We demonstrated that for the largest proportion of participants, a linear model best accounted for trust behaviour. In such a model, participants weigh their own judgement against the trusted agent, trial after trial, in a consistent fashion.

## Data Availability

The datasets generated and/or analysed during the current study (and analyses) are available in the OSF repository, https://osf.io/pwjmv/.

## Appendix

See Tables 3 and 4 and Fig. 6.

**Table 3:** Performance of participants recruited on campus, versus those recruited online via Prolific

	Phase	Source	Deviance A	Deviance B	Weight of Advice
Mean	pre	On campus	0.0287	0.0159	0.526
		Prolific	0.0322	0.0155	0.623
	adjusted	On campus	0.0272	0.0259	0.284
		Prolific	0.0309	0.0290	0.357
	post	On campus	0.0270	0.0179	0.429
		Prolific	0.0277	0.0181	0.495

The campus recruits achieved higher accuracy; however, the same pattern of results is observed in both groups

**Table 4:** Number of trials in each of the phases, completed by each participant depending on which of the three ‘Early’, ‘Middle’ and ‘Late’ conditions they were assigned to

	Practice	Pre	Adjusted	Post
Early	5	20	20	65
Middle	5	40	20	45
Late	5	60	20	25

In all blocks of the experiment 20% of the trials came without recommendations. Note we do not report findings with respect to the ‘Early’, ‘Middle’ or ‘Late’ conditions in this paper. The table is included for the sake of transparency
